# Hybrid Aortic Surgery: Clinical Outcomes and Techniques in Complex Aortic Pathologies

**DOI:** 10.7759/cureus.72200

**Published:** 2024-10-23

**Authors:** Muhammet F Saglam, Emrah Uguz, Kemal E Erdogan, Hüseyin Ü Erçelik, Murat Yücel, Mete Hidiroglu, Murat Canyigit, Erol Sener

**Affiliations:** 1 Cardiovascular Surgery, Ankara Yıldırım Beyazıt University Faculty of Medicine, Ankara, TUR; 2 Cardiovascular Surgery, Ankara Bilkent City Hospital, Ankara, TUR; 3 Interventional Radiology, Ankara Yıldırım Beyazıt University Faculty of Medicine, Ankara, TUR

**Keywords:** aortic aneurysm, aortic dissection, aortic pathologies, endovascular repair, hybrid aortic surgery

## Abstract

Objective: This study aimed to evaluate the clinical outcomes of hybrid aortic surgery in patients with complex aortic pathologies, with a focus on technical success, postoperative morbidity, and mortality.

Methods: A retrospective observational study was conducted on 24 patients who underwent hybrid aortic surgery between 2019 and 2024. Four surgical approaches were categorized on the basis of the pathological location: (1) median sternotomy with bypass from the ascending aorta to the branches of the aortic arch, followed by thoracic endovascular aortic repair (TEVAR); (2) cervical incision with left carotid-to-subclavian bypass, right carotid-to-left carotid bypass, or right carotid-to-left subclavian bypass and TEVAR; (3) median laparotomy with bypass from the iliac arteries to the superior mesenteric artery, renal arteries, and celiac trunk followed by endovascular aortic repair (EVAR); and (4) femoral incision with crossover bypass and EVAR. Data on demographics, preoperative diagnoses, surgical techniques, and postoperative outcomes were collected.

Results: No significant differences in demographic characteristics or comorbidities were found between the surgical groups. However, a significant association was observed between the location of the aortic pathology and the chosen surgical approach (p=0.019). Patients who underwent the femoral incision approach had a shorter hospital stay than those who did not (p=0.075). Postoperative complications were generally low across all groups; however, infection rates were higher at specific anatomical sites, such as the femoral incision area (p=0.002). No significant differences between the groups were observed in hospital or early mortality rates.

Conclusion: Hybrid aortic surgery is a safe and effective approach for treating complex aortic pathologies, particularly when traditional open or endovascular procedures are individually insufficient. Minimally invasive techniques, such as femoral incisions, offer advantages in reducing recovery time but may be associated with higher infection rates in isolated anatomical sites. However, these benefits are questioned, and further studies with larger populations are needed to validate these findings.

## Introduction

Aortic pathologies are conditions that involve structural weakening of the aorta, particularly leading to life-threatening complications such as aortic aneurysms and dissections. If left untreated, these conditions can result in rupture, significantly increasing the risk of mortality [1,2]. Aortic aneurysms and dissections are life-threatening conditions that can lead to severe complications if not promptly addressed, including sudden death. Aneurysms, characterized by abnormal enlargement due to the weakening of the aortic wall, present a significant risk of rupture over time, making them a critical vascular emergency that demands urgent intervention. Similarly, aortic dissections involve the tearing of the aortic wall, which can create a false lumen, disrupting blood flow and potentially leading to serious cardiovascular and systemic complications [3,4].

Aortic pathologies have traditionally been managed through open surgical techniques, which involve removing the damaged portions of the aorta and replacing them using synthetic grafts. However, these procedures are associated with higher rates of complications and mortality, particularly in elderly and high-risk patients. In recent years, less invasive alternatives have gained prominence, thanks to advances in endovascular techniques [5,6]. Endovascular aortic surgery, such as endovascular aneurysm repair (EVAR) and thoracic endovascular aneurysm repair (TEVAR), has become a viable alternative for treating aortic pathologies by minimizing surgical trauma. These minimally invasive methods offer patients shorter recovery periods and reduce the likelihood of postoperative complications [7,8].

However, in certain cases, neither open surgery nor endovascular techniques alone are sufficient to address the complexity of aortic pathologies [9,10]. This is particularly true in cases involving complex anatomical structures, such as the branching regions of the aorta, where both approaches must be used in combination. This need has driven the development of hybrid surgical procedures. Hybrid aortic surgery involves combining endovascular stenting with bypass procedures performed on large branches of the aorta that are anatomically challenging to reach using conventional methods [11,12]. For example, in arcuate aortic pathologies, both bypass from the ascending aorta to the arcuate branches and stent placement in the arcuate descending aorta are performed. Similarly, bypass procedures are performed in abdominal aortic pathologies to maintain the blood supply to visceral branches such as the celiac artery, superior mesenteric artery, and renal arteries, followed by endovascular stent placement.

This hybrid approach offers a valuable solution, particularly for patient groups with high surgical risk or those in whom endovascular procedures alone are insufficient. However, clinical experience with hybrid procedures remains limited, and further data are needed to support broader applications. Hybrid aortic surgery procedures performed in our clinic over recent years have contributed important insights into the efficacy and safety of this method [13,14]. In this study, a retrospective evaluation of hybrid surgical procedures for aortic pathologies, technical success rates, patient morbidity and mortality data, and long-term outcomes were analyzed.

In this context, the future role and advantages of hybrid surgery in the treatment of aortic pathologies will be better understood. In addition, the criteria for appropriate patient selection, technical difficulties, and complication management of hybrid procedures will shed light, and the need for further experience and research in this field will be emphasized.

## Materials and methods

Study design

This retrospective observational study aimed to evaluate the clinical and surgical outcomes of patients who underwent hybrid surgical procedures for aortic pathologies in our clinic between 2019 and 2024. The preoperative and postoperative clinical conditions and details of the patients' surgical procedures were retrospectively analyzed via the electronic data system. The study was conducted in accordance with the Declaration of Helsinki, and the necessary ethical approval was obtained from the Ankara City Hospital Clinical Research Ethics Committee (Approval number: TABED 1-24-521). Informed consent was obtained from all patients, and patient data were anonymized.

Patient selection

Patients aged 18 years and any gender who were diagnosed with aortic pathology (aneurysm, dissection, intramural hematoma, pseudoaneurysm, etc.) and who underwent hybrid surgery in our clinic were included in the study. Patients who did not meet the inclusion criteria involved patients, patients with missing data, patients who underwent emergency surgery, and patients who underwent other concurrent surgical procedures were excluded.

Surgical methods

Hybrid surgical procedures are divided into four main groups according to the pathologic segments of the aorta and the anatomical characteristics of the patients:

Bypass through a median sternotomy and TEVAR application, bypass (debranching) from the ascending aorta to the branches of the arch aorta to provide blood supply to the truncus brachiocephalicus, left carotid artery, and left subclavian artery, followed by endovascular stent placement in the aortic arch and descending aorta. In each case, bypasses were performed on the basis of the patient's anatomy rather than a full bypass to all three branches. This method was applied to patients with ascending aorta and arch pathologies [15].

Bypass and TEVAR through a neck incision: Left carotid-to-subclavian bypass, right carotid-to-left carotid bypass, or right carotid-to-left subclavian bypass were performed to maintain the blood supply, followed by endovascular stent placement in the aortic arch and descending aorta. This method is preferred, particularly for distal arch and descending aorta pathologies [16].

Bypass and EVAR via median laparotomy: In cases of diffuse aneurysms or dissections of the abdominal aorta, for which a bypass was performed from the iliac arteries to the celiac artery, superior mesenteric artery, and renal arteries, followed by placement of an abdominal aortic endovascular stent (EVAR). In some cases, this method was expanded to include the descending aorta in instances where the pathology involved that anatomical region [17].

Crossover bypass and EVAR through a femoral incision: In patients with iliac artery occlusion, crossover bypass was performed from the right femoral artery to the left femoral artery through bilateral incisions, followed by abdominal aortic EVAR. This method is particularly useful in cases where the vascular anatomy or the condition of the iliac arteries renders other options unsuitable [18].

Data collection and evaluation

Demographic information, preoperative diagnoses, intraoperative surgical techniques, and postoperative outcomes were collected through the hospital data processing system. Comorbidities such as hypertension, diabetes, smoking, chronic arterial disease, and chronic obstructive pulmonary disease (COPD) were also analyzed. The technical details of the surgical procedures, intraoperative and postoperative complications, length of hospitalization, and complication rates were recorded.

Statistical analysis

The data obtained were analyzed via SPSS 29.0 software (IBM Corp., Armonk, New York, USA). Categorical data are presented as frequencies and percentages, whereas numerical data are expressed as the means and standard deviations. The chi-square test was used to compare categorical variables, and the Kruskal-Wallis H test was used to analyze numerical data that were not normally distributed. In all analyses, p<0.05 was considered statistically significant.

## Results

Table 1 shows the demographic and clinical characteristics of patients undergoing different surgical approaches. As demonstrated, there were no statistically significant differences among the groups in terms of age, sex, hypertension, diabetes mellitus, coronary artery disease, smoking status, or chronic obstructive pulmonary disease (COPD) (p>0.05 for all variables).

**Table 1:** Demographic and clinical characteristics by surgical approach. COPD: chronic obstructive pulmonary disease, SD: standard deviation.

Variables	Median sternotomy (n=3)	Median laparotomy (n=5)	Cervical incision (n=14)	Femoral incision (n=2)	p-value	Mean ± SD
Age (years)	59.16 ± 14.42	59.16 ± 14.42	59.16 ± 14.42	59.16 ± 14.42	0.135	59.16 ± 14.42
Male	2 (66.7%)	4 (80%)	11 (78.6%)	1 (50%)	0.816	N/A
Female	1 (33.3%)	1 (20%)	3 (21.4%)	1 (50%)	N/A	N/A
Hypertension (Yes)	3 (100%)	4 (80%)	14 (100%)	2 (100%)	0.265	N/A
Hypertension (No)	0 (0%)	1 (20%)	0 (0%)	0 (0%)	N/A	N/A
Diabetes mellitus (Yes)	0 (0%)	0 (0%)	2 (14.3%)	0 (0%)	0.669	N/A
Diabetes mellitus (No)	3 (100%)	5 (100%)	12 (85.7%)	2 (100%)	N/A	N/A
Coronary artery disease (Yes)	1 (33.3%)	1 (20%)	2 (14.3%)	0 (0%)	0.778	N/A
Coronary artery disease (No)	2 (66.7%)	4 (80%)	12 (85.7%)	2 (100%)	N/A	N/A
Smoking (Yes)	1 (33.3%)	2 (40%)	10 (71.4%)	1 (50%)	0.474	N/A
Smoking (No)	2 (66.7%)	3 (60%)	4 (28.6%)	1 (50%)	N/A	N/A
COPD (Yes)	1 (33.3%)	0 (0%)	3 (21.4%)	0 (0%)	0.526	N/A
COPD (No)	2 (66.7%)	5 (100%)	11 (78.6%)	2 (100%)	N/A	N/A

Table 2 shows the distribution of aortic pathology types and postoperative outcomes across different surgical approaches. There were significant differences in the type and location of aortic pathology based on the surgical approach (p=0.019). Intraoperative and postoperative complications were minimal across all groups, with lower postoperative infection rates observed, particularly in the femoral incision group (p=0.002). Hospital mortality and early mortality rates did not significantly differ between the surgical groups (p>0.05). However, abnormal findings on control CT angiography were more prevalent in the sternotomy and laparotomy groups than in the other groups (p=0.050).

**Table 2:** Aortic pathology and postoperative outcomes by surgical approach. SD: standard deviation, chi-square test, p<0.05.

Variables	Median sternotomy (n=3)	Median laparotomy (n=5)	Cervical incision (n=14)	Femoral incision (n=2)	p-value
Aortic pathology type					
Abdominal aneurysm	1 (33.3%)	2 (40%)	2 (14.3%)	2 (100%)	0.285
Pseudoaneurysm	0 (0%)	1 (20%)	3 (21.4%)	0 (0%)	N/A
Dissection	2 (66.7%)	2 (40%)	9 (64.3%)	0 (0%)	N/A
Aortic pathology location					0.019
Abdominal aorta	1 (33.3%)	3 (60%)	1 (7.1%)	1 (50%)	
Thoracic aorta	0 (0%)	1 (20%)	4 (28.6%)	0 (0%)	N/A
Abdominal and thoracic aorta	2 (66.7%)	1 (20%)	9 (64.3%)	0 (0%)	N/A
Intraoperative complications					0.000
None	3 (100%)	5 (100%)	14 (100%)	2 (100%)	
Postoperative complications					0.000
None	3 (100%)	5 (100%)	14 (100%)	2 (100%)	
Hospital mortality					0.374
Yes	1 (33.3%)	0 (0%)	1 (7.1%)	0 (0%)	
No	2 (66.7%)	5 (100%)	13 (92.9%)	2 (100%)	N/A
Early morbidity					0.778
Yes	1 (33.3%)	1 (20%)	2 (14.3%)	0 (0%)	
No	2 (66.7%)	4 (80%)	12 (85.7%)	2 (100%)	N/A
Early mortality					0.063
Yes	1 (33.3%)	0 (0%)	0 (0%)	0 (0%)	
No	2 (66.7%)	5 (100%)	14 (100%)	2 (100%)	N/A
Postoperative infection					0.002
Yes	2 (66.7%)	0 (0%)	0 (0%)	0 (0%)	
No	1 (33.3%)	5 (100%)	14 (100%)	2 (100%)	N/A
Preoperative liver function tests					0.265
Normal	3 (100%)	4 (80%)	14 (100%)	2 (100%)	
Abnormal	0 (0%)	1 (20%)	0 (0%)	0 (0%)	N/A
Postoperative liver function tests					0.000
Normal	3 (100%)	5 (100%)	14 (100%)	2 (100%)	
Preoperative kidney function tests					0.374
Normal	2 (66.7%)	5 (100%)	13 (92.9%)	2 (100%)	
Abnormal	1 (33.3%)	0 (0%)	1 (7.1%)	0 (0%)	N/A
Postoperative kidney function tests					0.187
Normal	2 (66.7%)	4 (80%)	14 (100%)	2 (100%)	
Abnormal	1 (33.3%)	1 (20%)	0 (0%)	0 (0%)	N/A
Previous surgery					0.102
Yes	2 (66.7%)	4 (80%)	4 (28.6%)	0 (0%)	
No	1 (33.3%)	1 (20%)	10 (71.4%)	2 (100%)	N/A
Control CT angiography					0.050
Normal	2 (66.7%)	3 (60%)	11 (78.57%)	0 (0%)	
Abnormal	1 (33.3%)	2 (40%)	3 (21.43%)	0 (0%)	N/A
Reintervention					0.307
Yes	2 (66.7%)	1 (20%)	3 (21.4%)	0 (0%)	
No	1 (33.3%)	4 (80%)	11 (78.6%)	2 (100%)	N/A

Table 3 summarizes the ICU and ward stay durations by surgical approach. Although the length of ICU stay did not show statistically significant differences between the groups (p=0.331); however, there was a tendency towards shorter ward stays in the femoral incision group compared to the other approaches (p=0.075).

**Table 3:** Hospital stay by surgical approach. ICU: intensive care unit, SD: standard deviation.

Variables	Median sternotomy (n=3)	Median laparotomy (n=5)	Cervical incision (n=14)	Femoral incision (n=2)	p-value
ICU stay (days)	16.83 ± 1.00	10.30 ± 1.00	13.07 ± 1.00	7.50 ± 1.00	0.331
Ward stay (days)	18.00 ± 1.00	17.50 ± 1.00	10.21 ± 1.00	7.75 ± 1.00	0.075

## Discussion

This study aims to retrospectively evaluate the clinical outcomes of hybrid surgical procedures for aortic pathologies and compare the results between different surgical approaches. Hybrid surgery is a combination of open surgery and endovascular methods and is used in aortic segments that are anatomically difficult to access and risky. Especially in thoracic and abdominal aortic pathologies, hybrid approaches have become important treatment options with lower surgical trauma and complication rates when conventional surgical methods are limited [19].

In this study, no significant differences were found between demographic characteristics, comorbidities (hypertension, diabetes, coronary artery disease), and general health status regarding the surgical approach. This finding suggests that surgical decisions are based on the location and extent of aortic pathology rather than patient demographics. As shown in Table 2, more invasive surgical approaches, such as median laparotomy, were preferred for abdominal aortic pathologies, and median sternotomy was preferred for thoracic aortic pathologies. However, the femoral incision method, which is a minimally invasive approach, is frequently preferred for abdominal aortic aneurysms. These results suggest that surgeons consider anatomical factors when determining the surgical approach according to the location of the pathology [20,21].

When postoperative outcomes were evaluated, the advantages of minimally invasive surgical approaches became more evident. Notably, infection rates were lower in the femoral incision group, highlighting that such techniques may reduce the risk of infection, even in regions where higher infection rates are generally expected, such as the groin area [22]. As emphasized in the literature [23], minimally invasive surgical techniques are associated with fewer complications and lower infection rates. These results demonstrate that minimally invasive techniques facilitate faster recovery in the postoperative period.

One of the key findings of the study was the control CT angiography results. Patients who underwent median sternotomy and laparotomy had a higher rate of abnormal findings, suggesting that these more invasive approaches may increase the likelihood of incomplete surgical success, which could be reflected in complications detected on follow-up imaging. This observation supports the notion that minimally invasive methods might yield better long-term outcomes, positioning them as potentially safer alternatives for treating complex aortic pathologies [24].

No statistically significant difference was found between surgical methods in terms of hospital mortality rates and early mortality rates. These results suggest that hybrid surgery is a safe and effective option, even in high-risk patients. In addition, the hospitalization time was significantly shorter for procedures performed with minimally invasive techniques, such as femoral incisions, than those performed with other surgical methods [25]. This finding suggests that minimally invasive surgical techniques contribute positively to the recovery process of patients and allow for faster discharge.

This study has several limitations. First, as it was a retrospective study, the data were collected retrospectively, and some data may be missing. In addition, the limited sample size may affect the generalizability of the results. Larger, prospective studies are needed to confirm these results. Nevertheless, the findings are important in terms of demonstrating the clinical effects of hybrid surgery and the advantages of minimally invasive methods.

## Conclusions

In this study, hybrid surgical procedures demonstrated safety and efficacy in treating aortic pathologies, particularly with regard to the minimal complication rates observed across all surgical approaches. While minimally invasive techniques, such as femoral incisions, showed a trend toward shorter recovery times, these findings must be interpreted cautiously due to the small sample size. Larger, prospective studies are needed to further investigate the long-term outcomes and broader clinical implications of hybrid surgery, allowing for a more comprehensive evaluation of its benefits and potential limitations.

## References

[1] Özdemir-van Brunschot DM, Zerellari R, Tevs M, Wassiljew S, Holzhey D (2024). Multilayer flow modulator stent for aortic pathology: a meta-analysis and additional data from a single-centre retrospective cohort. Rev Cardiovasc Med.

[2] Ossola P, Mascioli F, Luzzi AP, Epis L, Coletta D (2024). Situs viscerum inversus and abdominal aortic aneurysm: a systematic review of a rare association. Intractable Rare Dis Res.

[3] Ilic N, Zlatanovic P, Petrovic F, Dragas M (2024). Prophylactic vacuum assisted abdominal wound closure versus primary abdominal wall closure after open repair of ruptured abdominal aortic aneurysm. Eur J Vasc Endovasc Surg.

[4] Desai ND, Wang GJ, Brinkman W (2024). Outcomes of a novel single branched aortic stent graft for treatment of type B aortic dissection. Ann Thorac Surg.

[5] AbuRahma AF, Avgerinos ED, Chang RW (2022). Society for Vascular Surgery clinical practice guidelines for management of extracranial cerebrovascular disease. J Vasc Surg.

[6] Koh BJ, Lee Q, Wee IJ (2022). Frailty scoring in vascular and endovascular surgery: a systematic review. Vasc Med.

[7] Lee WA, Matsumura JS, Mitchell RS (2011). Endovascular repair of traumatic thoracic aortic injury: clinical practice guidelines of the Society for Vascular Surgery. J Vasc Surg.

[8] Karaolanis GI, Makaloski V, Jungi S (2024). Endovascular repair of pararenal and thoracoabdominal aortic aneurysms with inner and outer off-the-shelf multibranched endografts: a systematic review and meta-analysis. J Vasc Surg.

[9] Rao MM, Zhong J, Mahay U, Haslam P, Patel J (2024). A national survey of interventional radiology trainees on peripheral and aortic endovascular training. Br J Radiol.

[10] Agarwal G, Hamady M (2024). Complex abdominal aortic aneurysms: a review of radiological and clinical assessment, endovascular interventions, and current evidence of management outcomes. BJR Open.

[11] Shi F, Wang Z (2020). Acute aortic dissection surgery: hybrid debranching versus total arch replacement. J Cardiothorac Vasc Anesth.

[12] Rana MA, Gloviczki P, Oderich GS (2012). Endovascular stenting with open surgery for reconstructions of the ascending aorta and the aortic arch: a review of indications and results of hybrid techniques. Perspect Vasc Surg Endovasc Ther.

[13] Kavanagh EP, Sultan S, Jordan F, Elhelali A, Devane D, Veerasingam D, Hynes N (2021). Hybrid repair versus conventional open repair for aortic arch dissection. Cochrane Database Syst Rev.

[14] Cao P, De Rango P, Czerny M (2012). Systematic review of clinical outcomes in hybrid procedures for aortic arch dissections and other arch diseases. J Thorac Cardiovasc Surg.

[15] Andacheh I, Lara G, Biswas S, Nurick H, Wong N (2019). Hybrid aortic arch debranching and TEVAR is safe in a private, community hospital. Ann Vasc Surg.

[16] Wooster M, Back M, Sutzko D, Gaeto H, Armstrong P, Shames M (2018). A 10-year experience using a hybrid endovascular approach to treat aberrant subclavian arterial aneurysms. Ann Vasc Surg.

[17] Sharma A, Sethi P, Gupta K (2020). Endovascular abdominal aortic aneurysm repair. Interv Cardiol Clin.

[18] Slappy AL, Hakaim AG, Oldenburg WA, Paz-Fumagalli R, McKinney JM (2003). Femoral incision morbidity following endovascular aortic aneurysm repair. Vasc Endovascular Surg.

[19] Harky A, Fok M, Bashir M (2020). Which is the optimal frozen elephant trunk? A systematic review and meta-analysis of outcomes in 2161 patients undergoing thoracic aortic aneurysm surgery using E-vita OPEN PLUS hybrid stent graft versus Thoraflex™ Hybrid prosthesis. Braz J Cardiovasc Surg.

[20] Naddaf A, Hasanadka R, Hood D, Hodgson K (2020). Repair of an anastomotic pseudoaneurysm with a novel hybrid technique. Ann Vasc Surg.

[21] Ali-Hasan-Al-Saegh S, Takemoto S, Shafiei S (2024). Sutureless aortic valve replacement with perceval bioprosthesis superior to transcatheter aortic valve implantation: a promising option for the gray-zone of aortic valve replacement procedures-a state-of-the-art systematic review, meta-analysis, and future directions. J Clin Med.

[22] Guo Y, Shao J, Shi W, Tian M, Li L, Ermek T, Zhang Z (2021). One-stop hybrid surgery for treatment of complex Stanford type B aortic dissection. J Int Med Res.

[23] Langer NB, Argenziano M (2016). Minimally invasive cardiovascular surgery: incisions and approaches. Methodist Debakey Cardiovasc J.

[24] Pham DT, Le TN, Doan HQ (2022). Innovative techniques of left atrial exposure in minimally invasive mitral valve repair. Asian Cardiovasc Thorac Ann.

[25] Alcocer F, Perez S, Martinez C (2012). Small skin incision and fistula elevation for hemodialysis using the femoral vein. J Vasc Surg.

